# Survival Benefit of Surgical Resection for Pancreatic Neuroendocrine Tumors With Oligometastatic Liver Metastasis: A Retrospective and Propensity Score-Matching Analysis

**DOI:** 10.3389/fonc.2022.903560

**Published:** 2022-06-30

**Authors:** Zhen Yang, Jie Liang, Kaiming Leng, Guangjun Shi

**Affiliations:** Department of Hepatopancreatobiliary Surgery, Qingdao Municipal Hospital, Qingdao University, Qingdao, China

**Keywords:** pancreatic neuroendocrine tumors, liver metastasis, surgery, survival, PSM

## Abstract

**Background:**

Pancreatic neuroendocrine tumors (PanNETs) are a heterogeneous group of pancreatic malignancies. Surgical resection is the only curative treatment option for patients with localized PanNETs, yet the role of cancer-directed surgery (CDS) in the setting of oligometastatic liver metastasis remains a controversy.

**Methods:**

All patients diagnosed with PanNETs and liver-only metastasis from 2010 to 2018 were identified from the SEER database. The biases of baseline characteristics between CDS and no-CDS cohorts were reduced by the propensity score-matching (PSM) method, and the prognostic role of CDS was estimated using the Kaplan–Meier method and Cox regression models. Logistic regression analysis was utilized to identify factors associated with patients who underwent CDS.

**Results:**

A total of 1,270 PanNET patients with oligometastatic liver metastasis were included and analyzed. Of these patients, 283 (22.3%) patients underwent CDS of the primary tumor, while the remaining 987 (77.7%) did not. The OS and CSS were significantly better in the CDS cohort regardless of the propensity score analysis. Multivariate analysis revealed that age, tumor differentiation, tumor location, and lymph node status were significantly associated with patients who were more likely to receive CDS.

**Conclusion:**

Our study demonstrated that CDS was associated with survival benefits in selected patients with PanNETs and liver-only metastasis based on a large population database.

## Introduction

Pancreatic neuroendocrine tumors (PanNETs) are a heterogeneous group of pancreatic malignancies arising from the islet cells of the pancreas, accounting for only 1%–2% of all pancreatic tumors (1–3). However, the annual incidence of PanNETs has been increasing dramatically over the past 40 years, owing primarily to the widespread use of cross-sectional imaging (4, 5). Unlike pancreatic ductal adenocarcinoma (PDAC), these tumors are commonly regarded as characteristically slow-growing neoplasms associated with a favorable prognosis. Indolent behaviors may, to some extent, delay diagnosis, such as when metastases, predominantly to the liver, are present in patients at diagnosis. Also, compared to other gastroenteropancreatic NETs, PanNETs are more frequently diagnosed at advanced stages with the presence of distant metastases (6–8). Approximately 80% of metastatic patients have secondary liver lesions, and in nearly 50% of patients, the liver is the only metastatic site. Although surgical resection is the only curative treatment option for patients with localized PanNETs, the role of primary tumor resection in the setting of oligometastatic disease remains controversial (9–13). In consideration that PanNET patients with metastatic disease may even derive survival benefits from debulking operations according to several studies, there is an urgent need to gather evidence for the benefits of cancer-directed surgery and identify clinicopathological factors that assist in the selection of candidates for primary tumor removal.

Therefore, in the present study, we aimed to use the SEER database to determine whether primary tumor resection could confer survival benefits in patients with hepatic oligometastatic PanNETs and to establish clinical criteria for selecting patients most likely to benefit from cancer-directed surgery.

## Materials and Methods

Records of patients with PanNETs between 2010 and 2018 were extracted and collected retrospectively from the SEER database, which covers nearly 30% of the population in the United States. The study was approved by the institutional review board (IRB) of Qingdao municipal hospital, and the informed consent was waived owing to the deidentified data source. The evaluated variables included patients’ demographics, tumor characteristics, treatment modalities, and survival outcomes. The inclusion criteria were as follows: (i) patients with histological confirmation of PanNET diagnosis, (ii) patients with liver-only metastases at the time of diagnosis, and (iii) patients with complete data on treatment and survival status. The exclusion criteria were set as: (i) patients with other metastatic sites such as bone, lung, and brain; and (ii) patients with missing information on treatment and oncological outcomes. The primary endpoint was overall survival (OS), which was defined as the interval from the initial treatment to death or the last follow-up time point.

### Statistical Analysis

Categorical variables were expressed as numbers (percentage) and compared using the Chi-square test. Univariate and multivariate logistic regression analyses were performed to determine the association between clinical variables and receipt of primary tumor resection. The overall survival (OS) and cancer-specific survival (CSS) between the different groups were compared using Kaplan–Meier estimates with log-rank tests. Univariate and multivariate Cox proportional hazard regression models were applied to identify prognostic factors associated with OS in all PanNETs and in PanNET patients who received cancer-directed surgery, respectively. Propensity score-matching (PSM) analysis was conducted to reduce the selection biases and confounding variables between the study cohorts (14, 15). All statistical analyses were performed using R software (version 4.3.2). A *p*-value of <0.05 was considered statistically significant. In addition, patients with missing values on any of the analyzed predictors were not included in the regression model.

## Results

### Baseline Characteristics

Between 2010 and 2018, a total of 1,270 patients with histologically confirmed PanNETs and hepatic metastasis at the time of diagnosis were identified and analyzed in our study. Of these patients, 283 (22.3%) underwent cancer-directed surgery (CDS) of the primary tumor, while the remaining 987 (77.7%) did not. Patient characteristics and clinicopathologic features are presented and compared in **Table 1** before and after PSM. As shown in **Table 1**, before PSM, patients who underwent CDS were significantly younger and tended to be classified as having a higher proportion of lymph node metastasis compared to those who did not undergo CDS. In addition, patients in the CDS cohort were more likely to have well-differentiated and functional tumors. After PSM, 176 patients were matched in each group, and the comparisons between the two groups showed that baseline characteristics were well-balanced.

**Table 1:** Comparison of baseline characteristics before and after propensity score matching.

Variables	Before PSM			After PSM		
	CDS group (*n* = 283)	No-CDS group (*n* = 987)	*p*-value	CDS group (*n* = 176)	No-CDS group (*n* = 176)	*p*-value
Gender			0.638			0.191
Male	159 (56.2%)	570 (57.8%)		100 (56.8%)	112 (63.6%)	
Female	124 (43.8%)	417 (42.2%)		76 (43.2%)	64 (36.4%)	
Age			**<0.001**			0.630
<65 years	216 (76.3%)	571 (57.9%)		127 (72.2%)	131 (74.4%)	
≥65 years	67 (23.7%)	416 (42.1%)		49 (27.8%)	45 (25.6%)	
Race			0.223			0.767
White	228 (80.6%)	781 (79.1%)		143 (81.3%)	140 (79.5%)	
Black	27 (9.5%)	127 (12.9%)		15 (8.5%)	19 (10.8%)	
Other	28 (9.9%)	79 (8.0%)		18 (10.2%)	17 (9.7%)	
Marital status			0.745			0.913
Married	169 (59.7%)	600 (60.8%)		108 (61.4%)	109 (61.9%)	
Other	114 (40.3%)	387 (39.2%)		68 (38.6%)	67 (38.1%)	
Tumor size			0.367			0.668
<2 cm	15 (5.3%)	56 (5.7%)		12 (6.8%)	10 (5.7%)	
2–4 cm	88 (31.1%)	349 (35.3%)		56 (31.8%)	50 (28.4%)	
≥4 cm	180 (63.6%)	582 (59.0%)		108 (61.4%)	116 (65.9%)	
Tumor grade			**<0.001**			0.348
Well differentiated	224 (79.2%)	269 (27.3%)		123 (69.9%)	122 (69.3%)	
Poorly differentiated	36 (12.7%)	107 (10.8%)		30 (17.0%)	23 (13.1%)	
Unknown	23 (8.1%)	611 (61.9%)		23 (13.1%)	31 (17.6%)	
Tumor location			0.090			0.197
Head	78 (27.6%)	321 (32.5%)		51 (29.0%)	56 (31.8%)	
Body/tail	146 (51.6%)	437 (44.3%)		87 (49.4%)	71 (40.4%)	
Other	59 (20.8%)	229 (23.2%)		38 (21.6%)	49 (27.8%)	
Functional status			**<0.001**			0.336
Functional	143 (50.5%)	323 (32.7%)		85 (48.3%)	76 (43.2%)	
Nonfunctional	140 (49.5%)	664 (67.3%)		91 (51.7%)	100 (56.8%)	
Lymph node metastasis			**<0.001**			1.000
No	100 (35.3%)	768 (77.8%)		100 (56.8%)	100 (56.8%)	
Yes	183 (64.7%)	219 (22.2%)		76 (43.2%)	76 (43.2%)	
Chemotherapy			**<0.001**			0.443
No	197 (69.6%)	440 (44.6%)		105 (59.7%)	112 (63.6%)	
Yes	86 (30.4%)	547 (55.4%)		71 (40.3%)	64 (36.4%)	

PSM, propensity score matching; CDS, cancer-directed surgery. Bold values indicate p < 0.05.

### Survival Outcomes Before and After PSM

**Figure 1:**
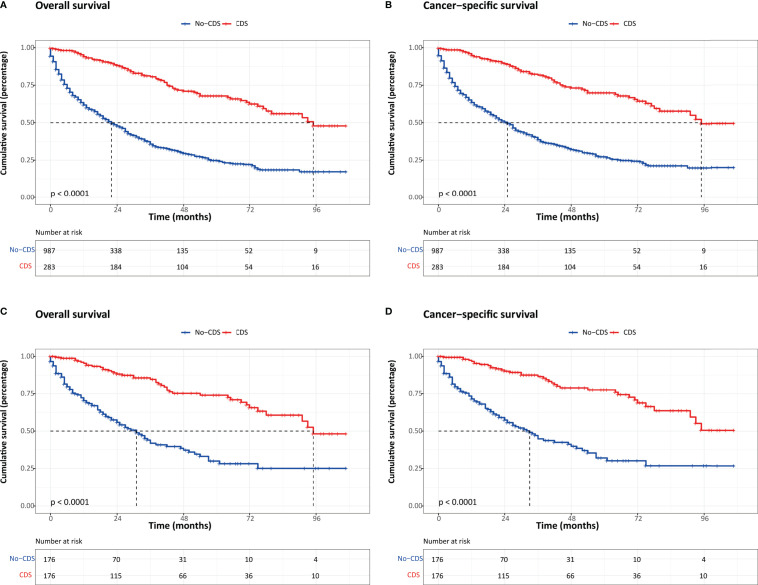
Kaplan–Meier survival estimates for patients in CDS and No-CDS groups: **(A)** overall survival before PSM. **(B)** Cancer-specific survival before PSM. **(C)** Overall survival after PSM. **(D)** Cancer-specific survival after PSM. CDS, cancer-directed surgery.

It is noteworthy that the overall survival (OS) and cancer-specific survival (CSS) were both significantly better in the CDS group than in the no-CDS group regardless of PSM or not. Before PSM, the median OS was 22.0 months in the no-CDS group and 95.0 months in the CDS group (*p* < 0.001). After PSM, the median OS was 95.0 and 31.0 months in the no-CDS and CDS cohorts, respectively (*p* < 0.001) (**Figure 1**).

### Subgroup Analysis of OS

**Figure 2:**
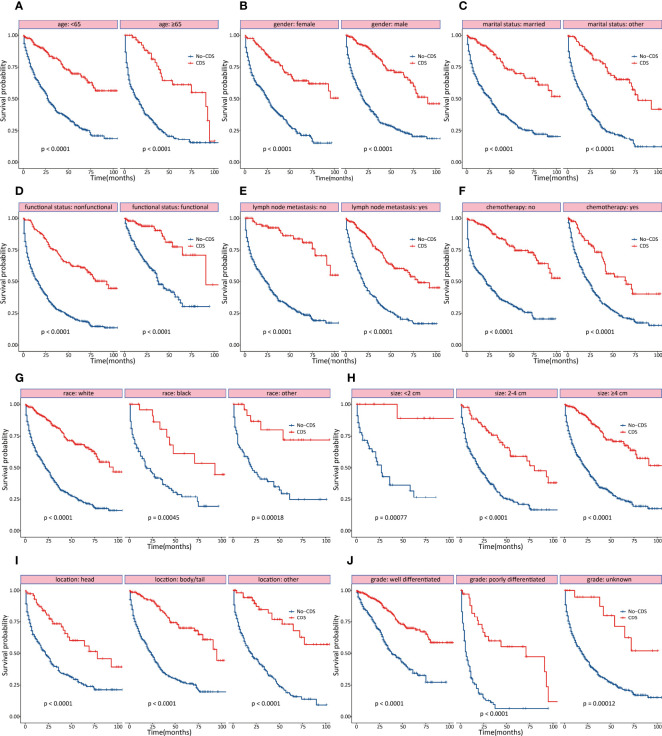
Subgroup analyses of OS stratified by clinicopathological characteristics. **(A)** age **(B)** gender **(C)** marital status **(D)** functional status **(E)** lymph node status **(F)** chemotherapy **(G)** race **(H)** tumor size **(I)** tumor location **(J)** tumor differentiation.

**Figure 3:**
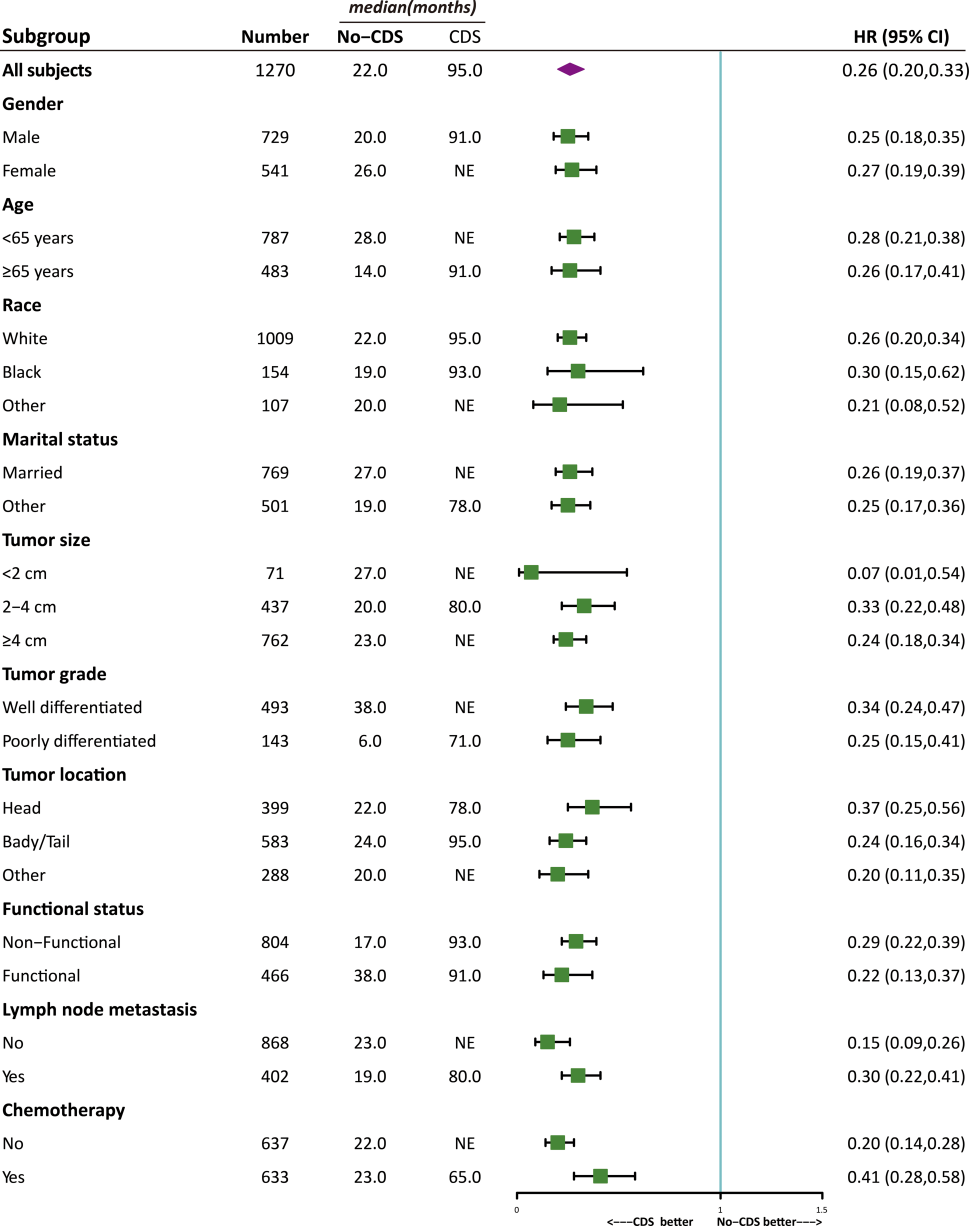
Effect of CDS on OS in all prespecified subgroups.

As shown in **Figures 2**, **3**, the effect of CDS on survival outcomes in all prespecified subgroups was then examined. After CDS, patients in the CDS cohort were associated with prolonged OS among all subgroups compared to those in the no-CDS cohort.

### Predictors of Survival

In multivariate COX regression analysis, when considering the whole population, age at diagnosis, marital status, tumor grade, functional status, and surgery were independently associated with OS (**Table 2**). As for patients who underwent CDS, multivariate analysis found that tumor grade, lymph node metastasis, and chemotherapy were independent prognostic factors for OS (**Table 3**).

**Table 2:** Association of clinicopathological factors with OS in oligometastatic liver metastasis in PNET patients.

Variables	Univariate analysis		Multivariate analysis	
	HR (95% CI)	*p*-value	HR (95% CI)	*p*-value
Gender				
Male	Ref			
Female	0.94 (0.81, 1.09)	0.419		
Age				
<65 years	Ref		Ref	
≥65 years	1.71 (1.47, 1.99)	**<0.001**	1.52 (1.30, 1.78)	**<0.001**
Race				
White	Ref			
Black	1.06 (0.85, 1.33)	0.614		
Other	0.90 (0.68, 1.19)	0.451		
Marital status				
Married	Ref		Ref	
Other	1.23 (1.06, 1.43)	**0.007**	1.31 (1.12, 1.52)	**0.001**
Tumor size				
<2 cm	Ref			
2–4 cm	1.42 (0.97, 2.06)	0.068		
≥4 cm	1.22 (0.84, 1.75)	0.294		
Tumor grade				
Well-differentiated	Ref		Ref	
Poorly differentiated	3.69 (2.90, 4.69)	**<0.001**	3.13 (2.44, 4.04)	**<0.001**
Tumor location				
Head	Ref			
Body/tail	0.86 (0.72, 1.02)	0.081		
Other	1.02 (0.84, 1.25)	0.830		
Functional status				
Nonfunctional	Ref		Ref	
Functional	0.46 (0.39, 0.56)	**<0.001**	0.58 (0.48, 0.70)	**<0.001**
Lymph node metastasis				
No	Ref		Ref	
Yes	0.80 (0.68, 0.94)	**0.006**	1.05 (0.89, 1.25)	0.552
Surgery				
No	Ref		Ref	
Yes	0.26 (0.20, 0.33)	**<0.001**	0.30 (0.23, 0.39)	**<0.001**
Chemotherapy				
No	Ref		Ref	
Yes	1.29 (1.11, 1.50)	**0.001**	0.90 (0.77, 1.06)	0.194

HR, hazard ratio. Bold values indicate p < 0.05.

**Table 3:** Univariate and multivariate analyses of OS in patients who underwent CDS.

Variables	Univariate analysis		Multivariate analysis	
	HR (95% CI)	*p*-value	HR (95% CI)	*p*-value
Gender				
Male	Ref			
Female	1.06 (0.67, 1.68)	0.799		
Age				
<65 years	Ref			
≥65 years	1.36 (0.81, 2.27)	0.247		
Race				
White	Ref			
Black	1.07 (0.53, 2.16)	0.861		
Other	0.76 (0.30, 1.89)	0.552		
Marital status				
Married	Ref			
Other	1.26 (0.80, 2.00)	0.323		
Tumor size				
<2 cm	Ref			
2–4 cm	6.40 (0.87, 47.00)	0.068		
≥4 cm	4.55 (0.63, 33.09)	0.135		
Tumor grade				
Well-differentiated	Ref		Ref	
Poorly differentiated	2.41 (1.40, 4.14)	**0.001**	1.87 (1.05, 3.32)	**0.032**
Tumor location				
Head	Ref			
Body/tail	0.61 (0.37, 1.02)	0.062		
Other	0.59 (0.31, 1.13)	0.110		
Functional status				
Nonfunctional	Ref		Ref	
Functional	0.50 (0.28, 0.88)	**0.016**	0.59 (0.33, 1.06)	0.076
Lymph node metastasis				
No	Ref		Ref	
Yes	2.22 (1.26, 3.91)	**0.006**	2.09 (1.18, 3.70)	**0.012**
Chemotherapy				
No	Ref		Ref	
Yes	2.18 (1.37, 3.46)	**0.001**	1.69 (1.03, 2.77)	**0.038**

CDS, cancer direct surgery; HR, hazard ratio. Bold values indicate p < 0.05.

### Factors Associated With CDS

To gain insight on patient selection, the logistic regression model was used to analyze the factors correlated with CDS (**Table 4**). On univariable analysis, age at diagnosis, tumor grade, tumor location, functional status, and lymph node status were found to be associated with patients, whether they received CDS or not. The significant factors in univariate analysis were then incorporated into the multivariate logistic regression model. Multivariate analysis demonstrated that those who were older than 65, with well-differentiated tumors, with tumors located in the pancreatic body or tail, and had lymph node metastasis were more frequently to undergo CDS. Among all the identified variables, lymph node status showed the most powerful association with CDS.

**Table 4:** Factors associated with PanNET patients who underwent CDS in the SEER database.

Variables	Univariate analysis		Multivariate analysis	
	OR (95% CI)	*p*-value	OR (95% CI)	*p*-value
Gender				
Male	Ref			
Female	1.07 (0.82, 1.39)	0.638		
Age				
<65 years	Ref		Ref	
≥65 years	2.35 (1.74, 3.18)	**<0.001**	1.92 (1.33, 2.78)	**<0.001**
Race				
White	Ref			
Black	0.73 (0.47, 1.13)	0.159		
Other	1.21 (0.77, 1.92)	0.404		
Marital status				
Married	Ref			
Other	1.05 (0.80, 1.37)	0.745		
Tumor size				
<2 cm	Ref			
2–4 cm	0.94 (0.51, 1.74)	0.847		
≥4 cm	1.16 (0.64, 2.09)	0.635		
Tumor grade				
Well-differentiated	Ref		Ref	
Poorly differentiated	0.40 (0.27, 0.61)	**<0.001**	0.48 (0.29, 0.79)	**0.004**
Tumor location				
Head	Ref		Ref	
Body/tail	1.38 (1.01, 1.88)	**0.044**	1.90 (1.28, 2.81)	**0.001**
Other	1.06 (0.73, 1.55)	0.762	1.12 (0.70, 1.79)	0.648
Functional status				
Nonfunctional	Ref		Ref	
Functional	2.10 (1.61, 2.75)	**<0.001**	1.52 (1.06, 2.19)	**0.023**
Lymph node metastasis				
No	Ref		Ref	
Yes	6.42 (4.82, 8.55)	**<0.001**	7.22 (5.09, 10.24)	**<0.001**

OR, odds ratio; CDS, cancer direct surgery. Bold values indicate p < 0.05.

### Exploratory Analyses

**Figure 4:**
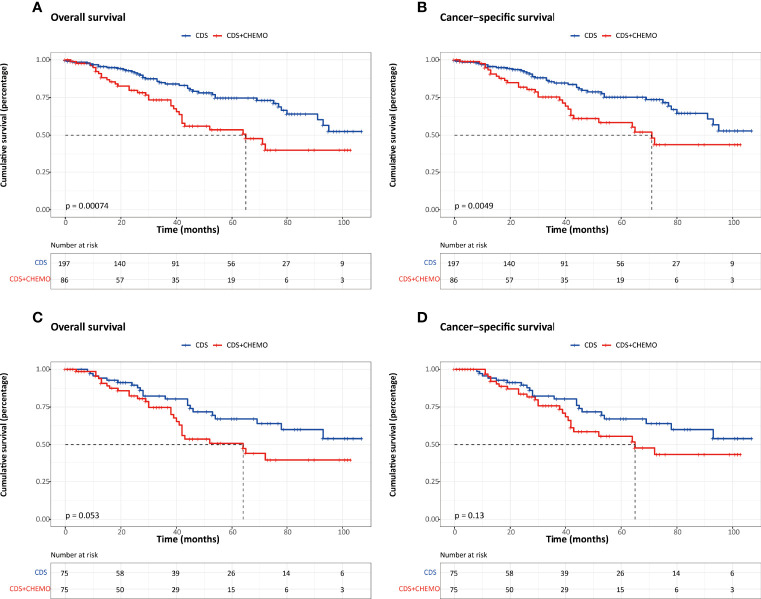
Kaplan–Meier survival estimates between patients who underwent CDS and those who underwent CDS and chemotherapy. **(A)** Overall survival before PSM. **(B)** Cancer-specific survival before PSM. **(C)** Overall survival after PSM. **(D)** Cancer-specific survival after PSM. CDS, cancer-directed surgery.

To compare the efficacy of chemotherapy, the survival outcomes were evaluated between patients who underwent CDS only and those who received CDS plus chemotherapy using the PSM method. Before PSM, we found that elderly patients (≥65 years), patients with well-differentiated, and functional tumors were more likely to undergo CDS alone (**Table 5**). Also, the OS and CSS were significantly better compared to those who underwent a combination of CDS and chemotherapy. After PSM, the baseline characteristics did not significantly differ between groups, and the survival outcomes were comparable (**Figure 4**).

**Table 5:** Comparison of baseline characteristics before and after propensity score matching.

Variables	Before PSM			After PSM		
	CDS (*n* = 197)	CDS + CHEMO (*n* = 86)	*p*-value	CDS (*n* = 75)	CDS + CHEMO (*n* = 75)	*p*-value
Gender			0.859			1.000
Male	110 (55.8%)	49 (57.0%)		43 (57.3%)	43 (57.3%)	
Female	87 (44.2%)	37 (43.0%)		32 (42.7%)	32 (42.7%)	
Age			**0.004**			1.000
<65 years	141 (71.6%)	75 (87.2%)		64 (85.3%)	64 (85.3%)	
≥65 years	56 (28.4%)	11 (12.8%)		11 (14.7%)	11 (14.7%)	
Race			0.539			0.681
White	158 (80.2%)	70 (81.4%)		57 (76.0%)	60 (80.0%)	
Black	21 (10.7%)	6 (7.0%)		8 (10.7%)	5 (6.7%)	
Other	18 (9.1%)	10 (11.6%)		10 (13.3%)	10 (13.3%)	
Marital status			0.486			0.320
Married	115 (58.4%)	54 (62.8%)		41 (54.7%)	47 (62.7%)	
Other	82 (41.6%)	32 (37.2%)		34 (45.3%)	28 (37.3%)	
Tumor size			0.324			0.861
<2 cm	12 (6.1%)	3 (3.4%)		3 (4.0%)	3 (4.0%)	
2–4 cm	65 (33.0%)	23 (26.7%)		23 (30.7%)	20 (26.7%)	
≥4 cm	120 (60.9%)	60 (69.8%)		49 (65.3%)	52 (69.3%)	
Tumor grade			**<0.001**			1.000
Well differentiated	167 (84.8%)	57 (66.3%)		57 (76.0%)	57 (76.0%)	
Poorly differentiated	15 (7.6%)	21 (24.4%)		10 (13.3%)	10 (13.3%)	
Unknown	15 (7.6%)	8 (9.3%)		8 (10.7%)	8 (10.7%)	
Tumor location			0.380			0.340
Head	51 (25.9%)	27 (31.4%)		18 (24.0%)	23 (30.7%)	
Body/tail	107 (54.3%)	39 (45.3%)		42 (56.0%)	33 (44.0%)	
Other	39 (19.8%)	20 (23.3%)		15 (20.0%)	19 (25.3%)	
Functional status			**0.001**			1.000
Functional	112 (56.9%)	31 (36.0%)		31 (41.3%)	31 (41.3%)	
Nonfunctional	85 (43.1%)	55 (64.0%)		44 (58.7%)	44 (58.7%)	
Lymph node metastasis			0.916			1.000
No	70 (35.5%)	30 (34.9%)		26 (34.7%)	26 (34.7%)	
Yes	127 (64.5%)	56 (65.1%)		49 (65.3%)	49 (65.3%)	

PSM, propensity score matching; CDS, cancer-direct surgery; CHEMO, chemotherapy. Bold values indicate p < 0.05.

## Discussion

A comparison of matched cohorts from the SEER database indicated that PanNET patients with liver-only metastasis who underwent CDS had significantly better survival outcomes compared to those without CDS. Our study also revealed tumor grade, lymph node metastasis, and chemotherapy were independent prognostic factors for OS in those who received CDS. In addition, we found that the combination of CDS and chemotherapy did not show survival advantages in comparison to CDS alone among the patients who performed CDS. To the best of our knowledge, it is the first study to verify the survival benefit of primary tumor resection focusing on PanNETs with oligometastatic liver metastasis based on a large population database from the United States.

According to the clinical presentation, PanNETs were classified as functional and nonfunctional tumors; of these, nonfunctional PanNETs accounted for nearly 80% (16, 17). Owing to a lack of specific symptoms and the indolent clinical course, a relatively large proportion of PanNETs were found to present with metastases at the time of diagnosis. Surgical resection is the mainstay treatment for patients with localized disease. However, issues regarding the management of PanNETs with metastases remain a subject of investigation, including the efficacy of primary tumor removal as well as the established criteria for selecting patients most likely to benefit from surgical resection (18, 19). Both the ENETS and NANETS guidelines currently do not recommend routine surgical resection in patients with metastatic PanNETs (20, 21). Other literature argued that neuroendocrine tumors have been regarded as one of few tumor types in which debulking operation could still yield survival benefits in metastatic disease (22, 23). Several previous studies have demonstrated the feasibility and safety of cancer-directed surgery in metastatic PanNET patients (24, 25). However, these publications only included small sample sizes of patients or analyzed patients with various metastatic sites. Our current study, on the other hand, included a large cohort of the population who were diagnosed with PanNETs and liver-only metastasis. A retrospective study including 882 patients with metastatic nonfunctioning pancreatic neuroendocrine tumors showed that removal of the primary tumor was associated with improved survival compared to those without resection (26). Feng et al. identified 350 patients with metastatic pancreatic neuroendocrine carcinoma and confirmed the survival benefits of primary tumor resection (27). Givi et al. investigated whether primary tumor resection in patients with gastrointestinal carcinoid neoplasm and hepatic metastases provides improved survival outcomes. A total of 84 patients were enrolled, 60 of whom received primary tumor resection, and survival analysis displayed that the resected group had a significantly longer median survival compared with the nonresected group (22). Compared with previous reports focusing on broader samples of metastatic PanNETs, our study differed in the respect that we concentrated specifically on patients with oligometastatic liver metastasis at diagnosis. Therefore, some differences may be attributed to the study population under analysis. In our study, we performed propensity score analysis to reduce the selection biases between groups and demonstrated the survival benefits of CDS even in the setting of liver-only metastatic disease. Furthermore, our results indicated that age at diagnosis, marital status, tumor grade, functional status, and surgery were independently associated with OS in all study populations.

In particular, multivariate logistic regression found that elderly patients, good tumor differentiation, tumors located in the body or tail of the pancreas, and lymph node metastasis were significantly correlated to patients who underwent CDS. In other words, these clinical variables might be used in patient selection when considering surgical treatment on PanNETs with liver-only metastasis. Subgroup survival analysis suggested that CDS was associated with survival advantages in all stratified groups, even in those with the presence of lymph node involvement. Owing to the indolent biological behavior, more aggressive surgical resection should be considered even in advanced stage patients.

Although there are various treatment options for PanNETs, surgery represents the cornerstone of the management because of the potential symptomatic and survival benefits (28–30). However, the role of chemotherapy in patients with liver metastasis remains unclear. In our study, we investigated the efficacy of chemotherapy in selected patients using the PSM method. In the matched cohorts, the survival results were comparable between patients who performed CDS alone and those who received CDS and chemotherapy.

Our study has some limitations. Firstly, the selection biases in this retrospective study cannot be fully avoided even though we used the PSM method. Secondly, the SEER database did not give us information on the extent of liver metastasis, such as the number and size of metastases. Thirdly, some important factors were not recorded in the SEER database, including the Ki-67 index, comorbidities, preoperative treatments, chemotherapy regimens, targeted therapy, and surgery-related data, which may influence our analysis. For example, patients with serious comorbidities were less likely to undergo surgery and were associated with poor survival results. Lastly, the definition of the functional status of PanNETs may not be quite accurate. Despite these limitations, our study sheds light on the role of CDS in oligometastatic liver metastatic PanNET patients.

## Conclusion

In conclusion, our study based on a large population database revealed that CDS was associated with survival benefits in selected patients with PanNETs and liver-only metastasis. It is imperative to keep in mind that the treatment option should be guided based on patient characteristics and interdisciplinary consultation.

## Data Availability Statement

The datasets presented in this study can be found in online repositories. The names of the repository/repositories and accession number(s) can be found below: https://seer.cancer.gov/.

## Author Contributions

GS and KL contributed to the conception and designed the study. ZY and JL drafted the manuscript and conducted the statistical analysis. All authors listed have made a substantial, direct, and intellectual contribution to the work and approved it for publication.

## Conflict of Interest

The authors declare that the research was conducted in the absence of any commercial or financial relationships that could be construed as a potential conflict of interest.

## Publisher’s Note

All claims expressed in this article are solely those of the authors and do not necessarily represent those of their affiliated organizations, or those of the publisher, the editors and the reviewers. Any product that may be evaluated in this article, or claim that may be made by its manufacturer, is not guaranteed or endorsed by the publisher.
